# Single-Lung Transplant Results in Position Dependent Changes in Regional Ventilation: An Observational Case Series Using Electrical Impedance Tomography

**DOI:** 10.1155/2016/2471207

**Published:** 2016-06-30

**Authors:** Kollengode Ramanathan, Hend Mohammed, Peter Hopkins, Amanda Corley, Lawrence Caruana, Kimble Dunster, Adrian G. Barnett, John F. Fraser

**Affiliations:** ^1^The Critical Care Research Group, The Prince Charles Hospital and the University of Queensland, Brisbane, QLD 4032, Australia; ^2^Queensland Centre for Pulmonary Transplant and Vascular Disease, The Prince Charles Hospital and the University of Queensland, Brisbane, QLD 4032, Australia; ^3^Institute of Health and Biomedical Innovation, Queensland University of Technology, 60 Musk Avenue, Kelvin Grove, QLD 4059, Australia

## Abstract

*Background*. Lung transplantation is the optimal treatment for end stage lung disease. Donor shortage necessitates single-lung transplants (SLT), yet minimal data exists regarding regional ventilation in diseased versus transplanted lung measured by Electrical Impedance Tomography (EIT).* Method*. We aimed to determine regional ventilation in six SLT outpatients using EIT. We assessed end expiratory volume and tidal volumes. End expiratory lung impedance (EELI) and Global Tidal Variation of Impedance were assessed in supine, right lateral, left lateral, sitting, and standing positions in transplanted and diseased lungs. A mixed model with random intercept per subject was used for statistical analysis.* Results*. EELI was significantly altered between diseased and transplanted lungs whilst lying on right and left side. One patient demonstrated pendelluft between lungs and was therefore excluded for further comparison of tidal variation. Tidal variation was significantly higher in the transplanted lung for the remaining five patients in all positions, except when lying on the right side.* Conclusion*. Ventilation to transplanted lung is better than diseased lung, especially in lateral positions. Positioning in patients with active unilateral lung pathologies will be implicated. This is the first study demonstrating changes in regional ventilation, associated with changes of position between transplanted and diseased lung.

## 1. Introduction

Single-lung transplants are becoming more common with the dearth of suitable lung donors. These patients have a challenging physiology with competing requirements of a diseased and a relatively nondiseased lung simultaneously after transplant. The native and transplanted lungs have variable compliance, which relate to their age/size and parenchymal structure. After transplant, all patients are evaluated with pulmonary function tests on a routine basis and in the event of any intervening illness. High resolution computerised tomography [CT] scan provides high resolution images which detects structural changes but allows only limited functional evaluation of lungs and has an unacceptable burden of radiation for routine use [1]. Scintigraphy and single-photon emission CT have been used to study individual lung function but spatial resolution is inadequate to provide useful assessment of regional lung volumes. Hyperpolarised helium-MRI [He MRI] has also been used to quantify split lung volumes and has been found to be superior to CT scans in determining relative effect of specific areas of lung participating in gas exchange. A worldwide shortage of He [2] and technical and financial constraints minimise the routine use of this technology [1].

Electrical impedance tomography (EIT) is a noninvasive, radiation-free bedside imaging technique of regional lung function. EIT is suitable for monitoring lung function at the bedside and is portable and noninvasive. It is a safe procedure because of the absence of requirement for ionising radiation and can be used in the vast majority of patients. Multiple aspects and trends of lung function including ventilation distribution, tidal volume, and functional residual capacity can be ascertained simultaneously.

Using EIT, we aimed to determine differences in global and regional ventilation between the diseased and transplanted lungs in single-lung transplant patients in five different positions. We hypothesised that the transplanted lung receives better ventilation than the native lung in all the five different positions.

## 2. Materials and Methods

This observational study was approved by the Human Resources Ethics Committee of the Prince Charles Hospital. Six patients after single-lung transplant were assessed using EIT at routine follow-up. Patients with single-lung transplant at any stage after transplant were invited to take part in the study over a 4-month period. All patients had a chest radiograph and pulmonary function test prior to EIT. Demographic data collected included age, sex, side of transplant, indication for transplant, and any relevant medical history.

### 2.1. Instrumentation

Images of ventilation distribution were obtained using the* EIT Evaluation Kit 2* supplied by Draeger* Medical Ltd.* with a temporal resolution of up to 50 images per second. The device is a functional two-dimensional device that uses a time-differencing algorithm (Newton-Raphson based algorithm) and generates “relative impedance change” data. EIT images reflect the lung function, not the lung itself, which means EIT displays ventilated lung regions rather than morphological or anatomical structures of the lung. There is a wide range in values for the resistivity of biological tissues and this can be dynamic. For example, the resistivity of the lungs changes very significantly during breathing, and many tissues change resistivity with blood volume changes during the cardiac cycle. The impedance changes measured by EIT can thus be an indicator of underlying organ function. Dynamic EIT images display relative changes in impedance with respect to a reference state; functional EIT images summarize these dynamic relative impedance changes over time. Impedance changes due to ventilation are normally about 10 times greater than impedance changes due to cardiac activity. Cardiac related impedance changes can quite easily be isolated using low pass filtering. EIT works on the principle that when an alternating current is injected between a pair of adjacent electrodes, it results in surface potentials that can be measured between the remaining adjacent electrode pairs. The surface potentials are then used to compute a sequence of impedance changes within the thorax using a reconstruction algorithm. The end expiratory lung impedance [EELI] is a measure of the functional residual capacity and the amplitude, which represents the tidal variation of impedance [TVar] that reflects the change in impedance from inspiration to expiration. This parameter correlates well with the tidal volume of the patient. Tidal variation of impedance only measures a cross section of the chest wall and while the relationship is predictive it does not measure tidal volume.

An EIT chest belt with sixteen electrodes was placed around the thorax of the patients in one transverse plane corresponding to the 5th intercostal space. A reference electrode was placed on patient's abdomen. We measured the EELI and the TVar in our six patients in the supine, right lateral, left lateral, sitting, and standing positions. Measurements were taken 15 minutes after change of position, to allow settling in the new position.

### 2.2. Statistical Instruments and Data Analyses

The collected data have repeated results from the same subjects and so cannot be analysed using standard statistical methods. Instead we used a mixed effects regression model with a random intercept for each subject. The random intercept controlled for the nonindependence in EIT results from the same subject. Differences of EELI and TVar were calculated between the transplanted and the native lung in each of the positions and results presented as mean differences with a 95% confidence interval [95% CI] and *p* value. A *p* value less than 0.05 was considered statistically significant.

## 3. Results

### 3.1. Demographics

There were more male patients and more patients who had left lung transplant (Table 1).

### 3.2. Tidal Variation to Each Lung in the Supine Position

The EELI results from the diseased lung were 670 arbitrary units [AU] lower on average (95% CI: –971, 211, *p* value = 0.21). The tidal variations in the diseased lung were 31% lower on average than the transplanted lung. However, the differences were not statistically significant in the supine position (Table 2).

### 3.3. Comparison of EELI and TVar between the Transplanted Lung and Native Lung in 5 Positions

**Figure 1:**
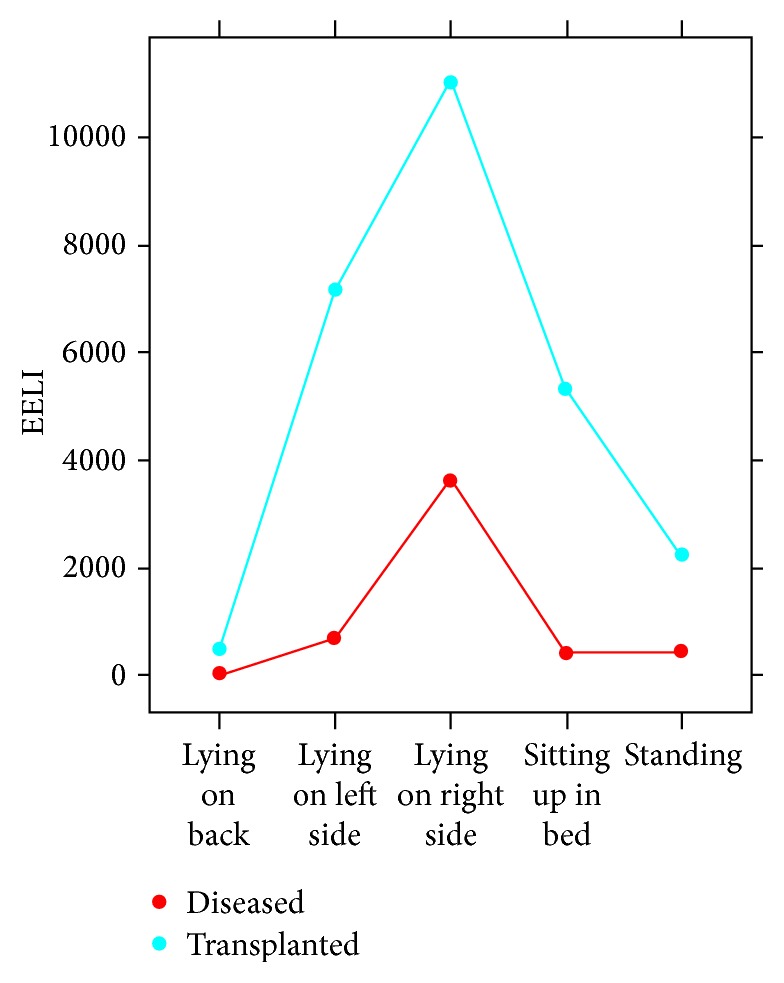
Trend of EELI changes in the various positions. The values are represented in arbitrary units.

The EELI was significantly lower in the diseased lung for lying on the left and right side (Table 3). The transplanted lung achieved more ventilation in the lateral positions. When lying on the right side the EELI in the diseased lung was 7395 AU lower on average than the transplanted lung. In the sitting position the diseased lung had less ventilation than transplanted lung, though not quite statistically significant (mean difference −5131 AU, CI −10531, 270, *p* value = 0.062). The mean EELI in the various positions is shown in Figure 1.

### 3.4. Results for Tidal Variation (%) by Position

**Figure 2:**
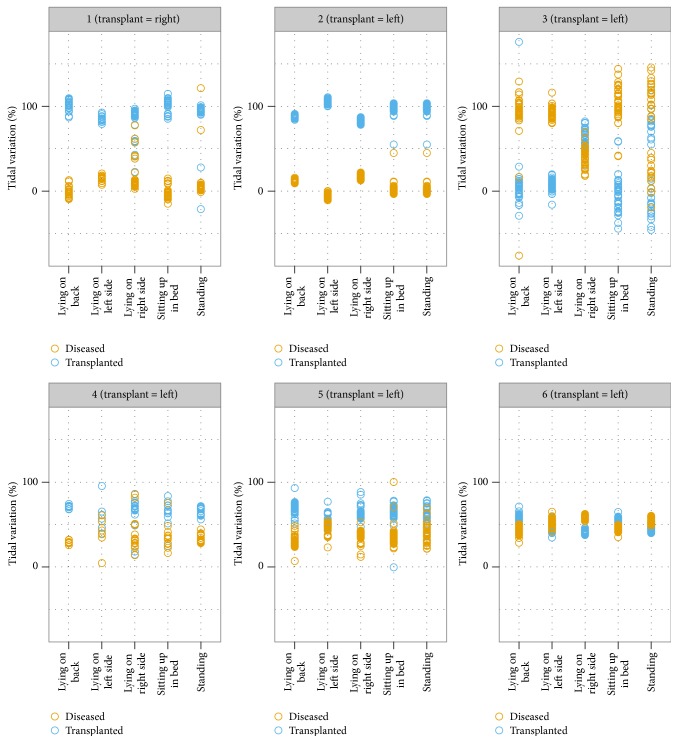
Plot of tidal variation [%] between diseased and transplanted lungs. Changes in tidal variation correlate with the tidal volume of the patient.

**Figure 3:**
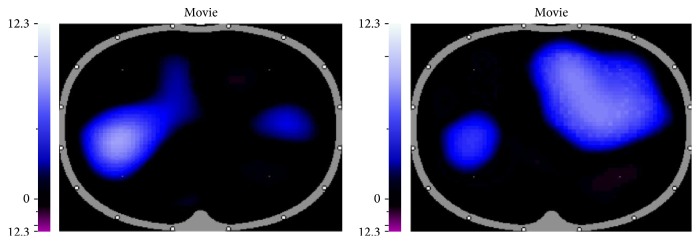
Pendelluft. It refers to asynchronous emptying of lungs. When the right lung is in the inspiration phase, the left lung starts to empty, and vice versa.

**Figure 4:**
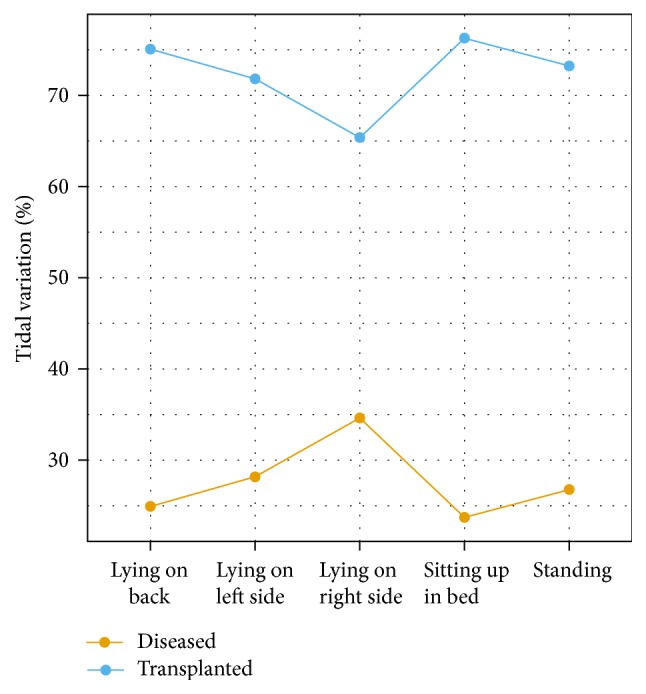
Differences for tidal variation without subject 3. The changes appear significant in all positions. Changes in tidal variation correlate with the tidal volume of the patient.

There were no statistically significant differences in tidal variation in any of the five positions assessed (Table 4). For most subjects the transplanted lung had a higher tidal variation. The notable exception was subject 3 who had a different plot for the tidal variation changes between transplanted and diseased lung (Figure 2). The images for the patient demonstrated pendelluft between the transplanted and diseased lungs (Figure 3). This asynchronous emptying of lungs in one patient strongly influenced the results of our small sample. The differences for the tidal variation became statistically significant for the majority of positions assessed when the data from this outlier was removed (subject 3) (Table 5, Figure 4).

## 4. Discussion

We assessed the regional ventilation in each lung using EIT in five different positions after single-lung transplantation. This study is the first of its kind in this cohort of patients. We noted that the transplanted lung always received better ventilation than the diseased lung in the supine as well as other positions. Ventilation between diseased and transplanted lung was significantly different in both the lateral positions after single-lung transplantation as determined using EELI and tidal variation values. Asynchronous emptying of lungs can lead to Pendelluft, which can lead to altered ventilation distribution between the lungs.

Lung transplantation is a viable treatment option for end stage lung disease. Single-lung transplants are becoming more common especially in nonseptic lung diseases [1]. Pulmonary function tests [PFT] and spirometry are routinely used for follow-up investigations after transplantation. However, these are highly variable and effort dependent. The utility of spirometry in this group of patient population is difficult to gauge as the tidal volumes and flows from the diseased lung will be added on to the ones from the transplanted lung. Spirometry will not give any added information about individual lung function in posttransplant patients. Any deterioration of the underlying disease makes it difficult to detect pathologies in the transplanted lung with this investigation modality. The deterioration of Fev1 and FVC can happen in a multitude of clinical conditions and hence both the parameters are neither sensitive nor specific tools for early detection of pathologies in the posttransplant period. High resolution CT [HRCT] scans and He MRI are capable of split lung analysis. Lobar and segmental morphological changes can be detected by HRCT and the investigation is quite sensitive as well. Density based segmentation enables calculation of lung volumes at CT and this can be performed individually for each lung. However there has been no correlation between the degree of functional impairment and the extent of morphological changes. Volumetry of lungs filled with He MRI correlates well with PFTs. However He MRI can be performed only in dedicated centres and hyperpolarised helium gas is not approved by Food and Drug Association for clinical use. Similarly, there is a worldwide shortage of this agent, and, in sick patients, helium inhalation may result in potentially dangerous hypoxemia [1].

EIT is a functional monitoring device used to study regional lung function. Its bedside use is safe as it does not involve radiation and it avoids transfer of critically ill patients to radiology suites. EIT is a technique based on the injection of small currents and voltage measurements using electrodes on the skin surface generating cross-sectional images representing impedance change in a slice of the thorax. While CT scanners typically provide images consisting of 512 × 512 pixels, EIT images from Draeger Kit only consist of 32 × 32 pixels, which are much less pixels compared to CT images. Even though the first CT scanner, developed in 1970, only provided an array of 80 × 80 pixels, it is not expected that the spatial resolution of EIT images can be improved in the near future to a point comparable with that of CT. In contrast to CT and MRI, the intended role of EIT in clinical practice is to guide therapy rather than the absolute diagnosis; it is unlikely that increased spatial resolution would significantly improve the ability of EIT to guide ventilation therapy.

The ability of EIT to study regional ventilation has been validated against many clinical reference methods including spirometry [3], inert gas washout technique [2], ventilation scintigraphy [4], single-photon emission [5], and electron beam computed tomography [6]. The change in regional ventilation with alteration of body position has been studied in both spontaneously breathing and anesthetised healthy subjects. In spontaneously breathing subjects, larger changes of air content occur in dependent regions in both inspiration and expiration. Hence dependent lung has better distribution of ventilation than the nondependent lung. Riedel et al. [7] evaluated the effect of body position and positive pressure ventilation on intrapulmonary tidal volume distribution in ten healthy adult subjects using EIT measurements and multiple-breath sulphur hexafluoride (SF6) washout. They observed asynchronous emptying of the dependent and independent lung in supine, prone, right, and left lateral positions. Rehder et al. [8] described that the independent lung receives more ventilation in anaesthetised, spontaneously breathing, and anaesthetised ventilated men. On the other hand, Frerichs et al. [9] used EIT analysis to show that the dependent lung is better ventilated in nonanaesthetised healthy subjects.

This study demonstrates that, in a group of patients who have had single-lung transplant, the ventilation was better in the transplanted lung than the diseased lung. Irrespective of whether the transplanted lung was dependent or nondependent when the patient was in the lateral position, the ventilation was significantly better in the transplanted lung. We believe that this might have clinical implications in positioning these patients for better distribution of ventilation and postoperative physiotherapy, when they develop unilateral lung pathologies. One patient demonstrated pendelluft with asynchronous emptying of the lungs. This refers to a hypothetical transfer of gas between alveoli of two different time constants. The ventilator inhomogeneity caused by pendelluft in our patient led to skewing of data in our cohort of patients.

The study has some limitations. The changes in impedance due to changes in blood volume of the heart and great vessels would have been isolated by low pass filtering in the kit. Also our study involved patients involved spontaneous breathing only and the results cannot be translated to mechanically ventilated patients. We also understand that EIT is a relatively new technology and research is still ongoing with respect to finding better variables of lung function.

This is the first study to demonstrate significant changes in regional ventilation associated with changes of position between transplanted and diseased lung. EIT is a technology with great bedside value and will have an important role to play in the future management of respiratory failure and rehabilitation of complex respiratory pathologies. The modality will be very useful both in the preoperative assessment of patients posted for single-lung transplantation and in the postoperative period, as a follow-up investigation to assess individual lung function. Further larger studies are needed to provide more insight into clinically relevant information in this cohort of patients.

## Figures and Tables

**Table 1:** Demographic details of the six subjects.

Male : female	5 : 1
Age	61.5 y
Right : left Tx	1 : 5
Baseline Spo2	>95%
Fev1 [range]	40–60%
Indication for Tx	
Pulmonary fibrosis	4
Emphysema	2

**Table 2:** EELI differences between transplanted lung and diseased lung in supine position. Negative results mean a greater reading in the transplanted lung.

Variable	Mean difference (diseased versus transplanted lung) [in AU]	95% confidence interval	*p* value
EELI	−697	−6102, 4707	0.80
Tidal variation (%)	−31	−83, 20	0.23

**Table 3:** EELI differences between transplanted lung and diseased lung in 5 positions. Negative results mean a greater reading in the transplanted lung.

Position	Mean difference (diseased versus transplanted lung) [in AU]	95% confidence interval	*p* value
Lying on back	−697	−6102, 4707	0.80
Lying on left side	−7065	−12472, −1657	0.011^*∗*^
Lying on right side	−7395	−12797, −1992	0.007^*∗*^
Sitting up in bed	−5131	−10531, 270	0.062
Standing	−2223	−7627, 3181	0.42

^*∗*^ *p* < 0.05, significant.

**Table 4:** Results for tidal variation (%) by position. Negative results mean a greater reading in the transplanted lung. Tidal variation of impedance measures only a cross section of the chest wall and correlates well with tidal volume.

Position	Mean difference (diseased versus transplanted lung) [in AU]	95% confidence interval	*p* value
Lying on back	−29	−74, 17	0.32
Lying on left side	−21	−66, 24	0.45
Lying on right side	−29	−74, 16	0.30
Sitting up in bed	−26	−71, 19	0.37
Standing	−30	−75, 16	0.30

**Table 5:** Differences for tidal variation without subject 3. Negative results mean a greater volume in the transplanted lung.

Position	Mean difference (diseased versus transplanted lung) [in AU]	95% confidence interval	*p* value
Lying on back	−52	−86, −17	0.003^*∗*^
Lying on left side	−45	−79, −10	0.012^*∗*^
Lying on right side	−31	−66, 3	0.076
Sitting up in bed	−54	−88, −19	0.002^*∗*^
Standing	−44	−78, −9	0.013^*∗*^

^*∗*^ *p* < 0.05, significant.
